# Norethindrone-Associated Transaminitis in Endometriosis Patients: A Case Series and Literature Review

**DOI:** 10.7759/cureus.67023

**Published:** 2024-08-16

**Authors:** Shawn Alexa Rosario, Emad Mikhail, Diana Encalada Soto

**Affiliations:** 1 Obstetrics and Gynecology, Morsani College of Medicine, University of South Florida, Tampa, USA

**Keywords:** progestin therapy, transaminitis, norethindrone, norethindrone / adverse effects, endometriosis

## Abstract

In this case series, we discuss 10 cases of norethindrone-induced transaminitis and conduct a literature review of this rare adverse event. A retrospective chart review was conducted on 10 patients (median age: 33 years) with diverse endometriosis phenotypes who received norethindrone and subsequently developed transaminitis, which is defined as elevated alanine transaminase (ALT) and aspartate transaminase (AST) levels. This condition was diagnosed in both asymptomatic and symptomatic patients, either during the work-up of acute symptoms or incidentally through routine lab tests. Our objective was to assess and characterize a case series of transaminitis associated with norethindrone use in endometriosis patients, detailing clinical presentations, management strategies, and outcomes. All cases exhibited normalization of liver function tests after discontinuation, occurring within one to 12 months with varying intervals of liver function testing. Patients receiving higher dosages (10 mg daily) demonstrated quicker resolution (average: four months). The reported adverse effects included nausea, vomiting, headache, rash, polyarthralgia, and abnormal uterine bleeding. Vigilant management, including prompt discontinuation, consistently resulted in the resolution of transaminitis. This study underscores the importance of continuous monitoring of liver function, even in asymptomatic patients on norethindrone therapy. Further investigations are imperative to identify specific groups susceptible to this adverse event.

## Introduction

Norethindrone acetate is a synthetic progestin-only contraceptive that plays a pivotal role in the medical management of endometriosis-associated chronic pelvic pain [1]. Hormonal therapies focusing on menstrual and ovulation suppression, particularly progestin-only contraceptives, are established treatments for symptomatic endometriosis [2]. Notably, when compared to both combined estrogen-progestin therapies and other progestin-only drugs, norethindrone has demonstrated comparable or superior patient satisfaction rates in alleviating endometriosis-associated symptoms [3,4]. While common adverse effects of norethindrone involve breakthrough bleeding, headaches, and mood changes, rare cases of elevated liver transaminase enzymes have been previously documented following its initiation [5-7]. In most reported cases, prompt withdrawal of norethindrone resulted in complete normalization of transaminase levels within weeks to months.

In this case series, we report 10 patients receiving treatment at an academic endometriosis center who subsequently presented with elevated alanine transaminase and aspartate transaminase levels, or transaminitis. The diagnosis was established either at the onset of symptoms indicative of liver injury confirmed via liver function testing, or incidentally during the routine lab work in asymptomatic patients. The primary objective of this report is to evaluate the clinical presentation and management of transaminitis in endometriosis patients undergoing norethindrone treatment.

## Materials and methods

A retrospective chart review was conducted on 10 patients who were found to have elevated liver enzymes after the initiation of norethindrone therapy for the treatment of endometriosis over a 10-year period. Patients were treated and followed by a subspecialized center for endometriosis care. Elevated liver enzymes are defined as alanine transaminase levels above 40 U/L or aspartate transaminase levels above 38 U/L. Each patient's clinical course was examined, including the initial presentation of endometriosis, patient-reported side effects, total duration of norethindrone therapy, and duration of norethindrone cessation until the resolution of transaminitis. We also assessed patients' biochemical profiles, reporting initial aspartate transaminase and alanine transaminase levels before and after the cessation of norethindrone. To validate our findings, we draw comparisons with published reports of norethindrone-induced transaminitis. Additionally, we explore the potential benefits of incorporating liver function monitoring into the long-term treatment strategy for patients receiving norethindrone.

## Results

The clinical and biochemical profiles of the presented patients are detailed in Tables 1, 2. The median age of patients in this study is 33 years, with ages ranging from 25 to 43 years. These cases describe patients who initially presented to a tertiary center specialized in complex gynecology and endometriosis care with dysmenorrhea, chronic pelvic pain, and/or abnormal uterine bleeding. Further imaging or laparoscopy revealed endometriosis, leading to the prescription of norethindrone to treat endometriosis-associated pain symptoms. The reported cases encompass diverse endometriosis phenotypes, including superficial peritoneal disease, ovarian endometriosis or endometriomas, and deep infiltrating endometriosis. Notably, six out of the 10 patients in this series reported improvement or complete resolution of their endometriosis-associated pain with the administration of norethindrone.

**Table 1:** Patient Demographics and Clinical Characteristics of Endometriosis AUB: abnormal uterine bleeding, BMI: body mass index (reference range: 19-25 kg/m^2^)

Case	Age (years)	Race/Ethnicity	BMI (kg/m^2^)	Clinical Presentation	Mode of Diagnosis	Endometriosis Phenotype
1	43	White	25.79	Pelvic pain, diarrhea	Laparoscopy	Deep infiltrating
2	27	African American	30.33	Pelvic pain, AUB	Laparoscopy	Deep infiltrating
3	31	African American	43.26	Pelvic pain, AUB	Imaging	Unspecified
4	35	White	21.52	Pelvic pain, AUB	Laparoscopy	Unspecified
5	30	White	29.02	Pelvic pain, AUB, dyspareunia	Laparoscopy	Superficial peritoneal
6	25	White	33.73	Pelvic pain, dyspareunia	Laparoscopy	Superficial peritoneal
7	26	Asian	21.60	Pelvic pain, dyschezia, dysuria	Laparoscopy	Deep infiltrating
8	37	White	22.58	Pelvic pain	Laparoscopy	Endometrioma
9	42	Hispanic/Latino	37.46	Pelvic pain, infertility	Laparoscopy	Superficial peritoneal
10	36	White	37.19	Pelvic pain	Imaging	Unspecified

**Table 2:** Biochemical and Clinical Profiles Before and After the Discontinuation of Norethindrone *Case 2: Patient was lost to follow-up.*Case 6: Follow-up CMP done at the annual exam. NE: norethindrone; COC: combined oral contraceptives; AUB: abnormal uterine bleeding; AST: aspartate transaminase (reference range: 12–38 U/L); ALT: alanine transaminase (reference range: 10–40 U/L); ALP: alkaline phosphatase (reference range: 25–100 U/L), TBil: total bilirubin (reference range: 0.1–1.0 mg/dL). Abnormal values are presented in bold.

Case	Dosage (daily, mg)	AST, initial (U/L)	ALT, initial (U/L)	ALP, initial (U/L)	TBil, initial (mg/dL)	AST, after NE cessation (U/L)	ALT, after NE cessation (U/L)	Total duration of treatment (mo)	Duration until Normalized LFTs (mo)	Reported adverse effects
1	5	214	27	32	1.9	17	23	7	1	None
2	5	580	1404	86	0.5	20	18	1	1	Nausea, vomiting
3	5	881	1752	60	1.3	23	25	6	1	Headache, nausea, vomiting
4	5	270	35	49	0.6	21	15	15	2	Headache, rash, polyarthralgia
5	5	37	69	73	0.8	24	25	4	2	None
6	5	37	63	60	0.5	18	28	8	2	None
7	5	31	49	49	0.5	21	24	19	3	AUB
8	10	66	40	48	0.4	15	11	7	4	AUB, dizziness
9	5	49	66	131	<0.1	25	17	11	12*	Mood changes
10	0.35	38	70	54	0.5	36	99	8	n/a*	AUB

Most patients were prescribed a standard effective dose of 5 mg daily of norethindrone, with one patient receiving 0.35 mg and another 10 mg daily. The average treatment duration was 8.6 months, ranging from one to 19 months (80%, n=8). In several cases, asymptomatic patients incidentally presented with elevated aminotransferase levels during follow-up visits. Patients experiencing symptomatic transaminitis reported symptoms such as nausea, non-bilious vomiting, rash, headache, and arthralgia without jaundice. In all the documented instances, norethindrone was promptly discontinued upon the identification of transaminitis. The cessation of the medication resulted in the normalization of levels and the resolution of associated symptoms, except for one case that was lost to follow-up. The time until resolution of transaminitis varied from one to 12 months, accounting for different monitoring intervals for transaminase levels. Following the discontinuation of norethindrone and the normalization of aminotransferase levels, four patients chose to switch to combined oral contraceptives, while three underwent surgical excision of endometriosis. Patients presented with varied phenotypes of endometriosis including superficial or peritoneal disease, deep infiltrating endometriosis, and ovarian endometriomas.

Among the cases where transaminitis resolved within one to two months after the discontinuation of norethindrone, three patients were initially symptomatic and admitted to the hospital for further work-up (Cases 2, 3, and 4). Two of these patients presented with the highest transaminase values, with initial aspartate transaminase/alanine transaminase (AST/ALT) ratios of 580:1404 and 881:1752 U/L (Cases 2,3; Table 2). The treatment duration with norethindrone in these patients ranged from one to 15 months.

Once elevated liver function tests (LFTs) were detected, patients underwent an extensive work-up with gastroenterology including serial liver function tests for acute hepatitis of unclear etiology, including Hepatitis B surface antigen, Hepatitis C antibody, Hepatitis A immunoglobulin M (IgM) antibody, Hepatitis E IgM antibody, cytomegalovirus, Epstein-Barr virus (EBV), Herpes Simplex Virus (HSV) 1 and 2, ceruloplasmin levels, and IgG and IgM measurements. Autoimmune and infectious work-up for all patients was largely negative with two exceptions; Case 2 had serology positive for EBV IgG and Case 4 was anti-nuclear antibody positive. Right upper quadrant abdominal ultrasound was obtained, and all cases demonstrated normal echotexture of the liver without evidence of mass or ductal dilatation. Notably, a liver biopsy was done in one patient following negative serologies, in which surgical pathology findings revealed no significant fibrosis, no steatohepatitis, and mild necro-inflammatory activity most consistent with drug-induced liver injury (Case 3). Two out of the three symptomatic patients received a course of oral corticosteroids during their hospital stay; however, this did not result in significant improvement of their transaminitis (Cases 3 and 4). Nevertheless, these patients with symptomatic transaminitis were found to have normalized AST/ALT levels within one to two months after discontinuing norethindrone.

Due to the variability of follow-up time of transaminases up to three months after the detection of elevation in AST and ALT levels, it is unclear if the transaminitis was prolonged or related to delay in follow-up testing. This group was largely asymptomatic, with a few patients reporting common side effects of norethindrone such as abnormal uterine bleeding, dizziness, and mood changes. These patients were followed on an outpatient basis with treatment durations ranging from eight to 19 months. While most of the patients in this study did not have baseline LFTs, one patient with a medical history of fatty liver disease had a prior measurement of normal LFTs prior to starting norethindrone (Case 9). However, this patient was not closely followed after the discovery of mild transaminitis; normalization of LFTs was observed a year later at an annual exam.

Notably, the patient with the longest treatment duration of 19 months was found to have normal liver transaminases at eight months mid-treatment. Subsequently, this patient developed asymptomatic transaminitis almost one year later, despite no changes to the medication regimen (Case 8). Like other reported cases, a complete resolution of transaminitis occurred on discontinuing norethindrone. These findings may suggest the need for continuous monitoring of liver enzymes in patients taking norethindrone despite normal prior testing. Given the absence of specific guidelines for monitoring liver function through transaminase levels in patients taking norethindrone, transaminitis was initially identified during work-up of symptomatic patients or incidentally in asymptomatic patients.

## Discussion

This case series outlines 10 cases of elevated transaminases due to suspected drug-induced liver injury (DILI) from norethindrone treatment in patients with endometriosis. DILI can result from the direct hepatotoxic effects of a drug or its reactive metabolite; parenchymal cell injury triggers immune cell activation, leading to the production of proinflammatory and hepatotoxic factors [8, 9]. The exact mechanism of liver injury in the context of progestin therapy, especially norethindrone, remains elusive. Derived from nortestosterone, which exhibits some androgenic activity, norethindrone has been tentatively linked to immune-mediated hepatotoxicity at the level of Kupffer cells [10, 11].

The initial assessment for suspected DILI involves measuring serum levels of AST, ALT, alkaline phosphatase (ALP), and total bilirubin. Patients with acute hepatitis, indicated by elevated serum aminotransferases, may be asymptomatic contingent on the injury severity [12]. According to the American Association for the Study of Liver Diseases (AASLD) guidelines, managing transaminitis due to suspected DILI necessitates the discontinuation of the causative agent and further investigation into other potential causes, including infectious or autoimmune etiologies [8]. The use of steroids in treating DILI remains controversial but may be considered if an underlying autoimmune process is suspected [13].

Only nine other cases of suspected norethindrone-associated transaminitis have been previously reported in the literature. One case involved a patient who developed abdominal pain, vomiting, and transaminitis following norethindrone initiation for excessive vaginal bleeding due to a fibroid [5]. Despite normal prior LFTs, AST and ALT levels were significantly elevated one month after commencing norethindrone, normalizing within two weeks of cessation. Notably, this patient had a history of fatty liver disease and a BMI of 36.3 kg/m^2^. Another series reported two patients of cholestatic jaundice due to norethindrone, one of which had a prior history of jaundice induced by combined oral contraceptives [6]. Liver transaminases normalized within four months after prompt norethindrone withdrawal and a course of oral prednisone. Two reports described six other documented cases in which transaminitis was found incidentally and spontaneously resolved after cessation of norethindrone [7, 14].

These reports suggest potential risk factors for norethindrone-induced transaminitis, including obesity, prior non-alcoholic liver disease, interactions with other hepatotoxic medications, and a history of liver injury from other hormonal treatments. Only one patient in our study had a known history of fatty liver disease, while five patients were classified as obese (BMI>30 kg/m^2^) and one was overweight (BMI 25-29.9 kg/m^2^). Studies have shown that elevated liver transaminases can be associated with higher BMI, potentially exacerbating the risk of drug-induced liver injury [15]. This observation highlights the need for further research to understand how these factors contribute to the development of transaminitis in patients receiving norethindrone or other progesterone-containing drugs.

Considering the timing of the earliest incidence of transaminitis in our cases and in prior literature, elevated aminotransferase levels may be measured as early as two weeks to one month after initiating norethindrone. Our cases highlight norethindrone-associated transaminitis in patients receiving dosages ranging from 0.35 mg to 10 mg daily for a duration of up to 19 months, with the most severe case occurring at a 10 mg dose daily for only one month. It remains unclear whether the dose or duration of norethindrone therapy influences the severity of transaminitis, as it appears to develop across various treatment regimens. The possibility of an idiosyncratic reaction also warrants consideration.

Previous case reports align with our findings regarding the complete resolution of transaminitis following norethindrone discontinuation. One case series describes asymptomatic transaminitis in three patients, resolving spontaneously upon discontinuation of norethindrone within two weeks [7]. Uneventful recovery after withdrawal of the drug seems to occur without additional interventions regardless of treatment duration, dosage, or patient's age. Interestingly, the additional use of prednisone in two of our cases did not result in improvement in transaminitis, which occurred after the cessation of norethindrone.

Our case series represents the largest reported in the current literature concerning norethindrone-associated liver injury. A few limitations of our study include the small sample size of patients and the retrospective nature of the data collection. This can be attributed to the rarity of this adverse event. Consequently, the generalizability of our findings may be limited. Additional prospective research is needed to fully understand progestin-mediated liver injury in humans and identify factors predisposing individuals to this condition. Possible risk factors, such as obesity and a prior history of liver disease, may be further explored in prospective studies that include baseline measurements of liver transaminases to establish a clear association between norethindrone and transaminitis.

A recent survey of a group of gynecologists revealed that most do not routinely monitor liver function in patients prescribed norethindrone [14]. Notably, only 20% of the surveyed gynecologists had previously diagnosed possible norethindrone-induced liver injury [14]. While progestin-induced transaminitis is uncommon, monitoring liver enzymes in patients taking norethindrone at any dose may be advisable, even in the absence of symptoms.

## Conclusions

This is the largest case series describing norethindrone-associated transaminitis. Our findings underscore the importance of monitoring liver enzymes in patients taking norethindrone, considering that not all individuals will experience liver injury. Elevated BMI may be a risk factor, though this needs to be further delineated in future studies. Surveillance of liver enzymes, including AST and ALT, may be especially crucial in patients with endometriosis, given the often-indicated long-term therapy due to the chronic nature of the disease.

Based on our observations, baseline liver function tests at the start of norethindrone therapy may be considered, with follow-up assessments at three months and then yearly. This testing interval is suggested to balance the need for early detection of liver injury with the lack of a uniform timeline for adverse effects. However, more research is needed to refine these recommendations and identify specific risk factors. Further studies should explore the relationship between elevated BMI and norethindrone-induced liver injury and determine more precise monitoring intervals to ensure patient safety.
